# Modulatory effects of transcranial direct current stimulation on sensory gating in Fibromyalgia Syndrome

**DOI:** 10.3389/fpsyg.2025.1607317

**Published:** 2025-08-19

**Authors:** Juan L. Terrasa, Christine Winterholler, Pedro Montoya, Antonio Juan, Casandra I. Montoro

**Affiliations:** 1 Cognitive and Affective Neuroscience and Clinical Psychology, Research Institute of Health Sciences (IUNICS) and Balearic Islands Health Research Institute (IdISBa), University of the Balearic Islands (UIB), Palma, Spain; 2 Rheumatology, Hospital Universitari Son Llàtzer, Palma de Mallorca, Spain; 3 Department of Psychology, University of Jaén, Jaén, Spain

**Keywords:** transcranial direct current stimulation, somatosensory cortex, sensory gating, late positive complex, Fibromyalgia Syndrome

## Abstract

**Introduction:**

Several studies have demonstrated a reduced habituation to redundant somatosensory stimulation (sensory gating) in Fibromyalgia Syndrome. Furthermore, anodal transcranial direct current stimulation has been shown to modulate somatosensory processing. The aim of this study was to examine the modulatory effects of anodal transcranial direct current stimulation applied over the left primary somatosensory cortex on sensory gating in Fibromyalgia Syndrome.

**Methods:**

Thirty-nine female Fibromyalgia Syndrome patients (43–71 years, mean 55.56 ± 7.85) participated in the study and were randomly assigned to the active transcranial direct current stimulation (*n* = 17) or non-electrical stimulation (sham; *n* = 22). Before and after transcranial direct current stimulation, somatosensory evoked potentials were recorded during a paired-pulse paradigm, consisting of two identical somatosensory stimuli (S1 and S2) applied in the right forefinger in rapid succession.

**Results:**

Whereas P50 and N100 components were unaltered, a significant modulatory effect on the difference S1 – S2-which is commonly considered an index of sensory gating-in the Late Positive Complex component was found. This modulation manifested as an increased difference in the right hemisphere (contralateral to the stimulation) and a decreased difference in the left hemisphere (ipsilateral).

**Discussion:**

Although this lateralizing pattern remains to be clarified, present results suggest brain excitability and somatosensory processing modulation by using anodal transcranial direct current stimulation in Fibromyalgia Syndrome patients.

## 1 Introduction

Fibromyalgia Syndrome (FMS) is a condition characterized by chronic widespread musculoskeletal pain with a global prevalence between 0.2 and 6.6% (Marques et al., 2017; Sarzi-Puttini et al., 2020). The syndrome is complex, involving symptoms like chronic pain, fatigue, cognitive dysfunction, sleep disturbances, hypersensitivity to pain (hyperalgesia/allodynia), and psychiatric conditions such as anxiety and depression (Chinn et al., 2016; Wolfe et al., 2010; Zhu et al., 2017). Additionally, FMS patients experience abnormal somatosensory information processing, including impaired sensory gating—a central nervous system mechanism that filters irrelevant sensory inputs (Montoya et al., 2006). This process prevents higher cortical regions from being overwhelmed with repetitive stimuli (Boutros et al., 1995; Ambrosini et al., 2001; Boutros and Belger, 1999).

Sensory gating is often studied using somatosensory evoked potentials (SEPs) in paired-pulse paradigms. These paradigms involve two rapid stimuli (S1 and S2), and sensory gating is assessed by analyzing the suppression of responses to the second stimulus (Boutros et al., 1995; Boutros and Belger, 1999; Boutros et al., 2009). This suppression is typically quantified as either the difference in SEP amplitudes between S1 and S2 (S1-S2) or the ratio of amplitudes elicited by S2 relative to S1 (S2/S1) (Fuerst et al., 2007). SEPs include components such as P50, N100, and the Late Positive Complex (LPC), which are measured via EEG. The P50 component, originating from the primary somatosensory cortex, is linked to early stimulus processing and emerges 50 milliseconds after stimulation (Eisenstein and Eisenstein, 2006; Hämäläinen et al., 1990; Desmedt and Tomberg, 1989). N100, generated in the secondary somatosensory cortex, appears around 100 milliseconds post-stimulus and is involved in attention and sensory-motor responses (Staines et al., 2014; Allison et al., 1992; Giard et al., 1988). The LPC, peaking approximately 300 milliseconds after stimulation, reflects higher-order cognitive processes such as decision-making and memory updating (Shen et al., 2020; Rushby et al., 2005). In healthy individuals, P50, N100, and LPC amplitudes decrease in response to the second stimulus, indicating effective sensory gating. However, FMS patients exhibit a failure to suppress these responses, suggesting impaired filtering of irrelevant stimuli (Montoya et al., 2006; Lijffijt et al., 2009).

Given this sensory dysfunction, there is growing interest in non-invasive neuromodulation techniques, particularly transcranial direct current stimulation (tDCS), as a potential treatment for FMS-related pain (Montoya et al., 2005). tDCS applies a low-intensity current (1–2 mA) to the cortex via electrodes, modulating neuronal excitability by altering resting membrane potentials without inducing action potentials directly (Lloyd et al., 2020; Thair et al., 2017; Lefaucheur et al., 2017). Anodal stimulation is commonly assumed to increase the likelihood of neuronal firing and overall cortical responsiveness, while cathodal stimulation decreases it (Lloyd et al., 2020; Thair et al., 2017). tDCS has been shown to influence brain functional connectivity (Nitsche et al., 2008; Cummiford et al., 2016; Neeb et al., 2019), leading to neurophysiological and psychological changes (Volz et al., 2016; Antal et al., 2010).

Specifically, anodal tDCS targeting the primary somatosensory cortex (SI) can induce changes throughout the somatosensory processing hierarchy, affecting both early sensory and late cognitive stages, including decision-making (Fujimoto et al., 2014; Hilgenstock et al., 2016; Hirtz et al., 2018; Labbé et al., 2016; Ragert et al., 2008). Studies have demonstrated the therapeutic benefits of tDCS for modulating somatosensory function in healthy participants (Vaseghi et al., 2015; Antal et al., 2008; Koyama et al., 2017) and in both acute (Borckardt et al., 2011) and chronic pain patients (Kikkert et al., 2019; Pollonini et al., 2020; Rahimi et al., 2020). Despite the extensive evidence of altered somatosensory processing in FMS, no study has yet examined the effects of tDCS on sensory gating in this population. This gap underscores the need for further research exploring how tDCS might address the sensory gating deficits characteristic of FMS.

In this sense, a previous study by our group demonstrated that anodal tDCS over the somatosensory cortex enhanced the suppression of the LPC component of SEPs during a sensory gating paradigm, indicating a modulation of late-stage inhibitory mechanisms (Montoro et al., 2021). Specifically, we observed a greater attenuation of LPC amplitudes in response to the second stimulus (S2) following stimulation—reflecting improved gating at late cortical processing stages—without significant effects on earlier components such as P50 and N100. The inability to inhibit, or “gate,” irrelevant sensory inputs has been associated with sensory and information overload, potentially leading to neuronal hyperexcitability due to disrupted habituation mechanisms, as observed in conditions such as schizophrenia (Vlcek et al., 2014). Although anodal stimulation is typically regarded as excitatory, our findings suggest that anodal tDCS may enhance top-down inhibitory control over redundant somatosensory input. While the traditional stimulation-dependent model of tDCS posits excitatory effects for anodal and inhibitory effects for cathodal stimulation, emerging evidence points to more complex activation–inhibition patterns (Jacobson et al., 2012). It emphasizes that the effects of tDCS are strongly shaped by ongoing network dynamics (Jacobson et al., 2012; Antal et al., 2014; Thirugnanasambandam et al., 2011; Woods et al., 2016), indicating that tDCS does not directly generate activity in resting neuronal networks but rather modulates existing patterns of spontaneous neuronal activity (Fritsch et al., 2010).

Therefore, the aim of this study was to examine whether anodal tDCS over the left SI can modulate sensory gating—measured by the S1-S2 difference—processing in FMS patients. Considering the reviewed bibliography, it was hypothesized that anodal tDCS would modify SEPs amplitude patterns (P50, N100 and LPC), resulting in attenuated cerebral responses to irrelevant stimulation in FMS patients. Furthermore, we will analyze the potential effect of tDCS on each hemisphere, considering that the stimulation used to induce sensory gating was applied only to the right hand and not bilaterally.

Importantly, this study did not aim to evaluate the clinical efficacy or long-term outcomes of tDCS. Instead, it focused on the basic neurophysiological mechanisms underlying inhibitory control—particularly the S1–S2 difference, which is commonly associated with somatosensory gating—and their modulation following a single session of anodal tDCS. This mechanistic approach is intended to enhance our understanding of the modulation of altered cortical excitability and sensory processing in FMS, thereby providing a foundation for future studies exploring potential links between tDCS, sensory gating, and clinical outcomes in this population.

## 2 Materials and methods

### 2.1 Participants

*A priori* power analysis indicated that a minimum sample size of 34 participants was required to achieve 80% statistical power, assuming an alpha level of 0.05 and an effect size of *f* = 0.25 (approximately equivalent to Cohen’s *d* ≈ 0.5–0.6, indicating an intermediate effect). Based on this, thirty-nine female volunteers (aged 43–71 years, mean 55.56 ± 7.85) diagnosed with Fibromyalgia Syndrome (FMS) participated in the study. Recruitment occurred through rheumatology units at Son Llátzer and Son Espases hospitals, the Balearic Fibromyalgia Support Association (ABAF), and the Association of Fibromyalgia, Chronic Fatigue Syndrome and Multiple Chemical Sensitivity of Inca and Comarcas (AFIC) in Spain. Eligibility required a confirmed FMS diagnosis by a rheumatology specialist at least 1 year prior, right-handedness, and absence of neurological, psychiatric, or cardiovascular conditions, tDCS contraindications, substance abuse history, or pharmacological treatments affecting the cardiovascular or central nervous systems. Despite this, 89.7% used analgesics, 87.2% antidepressants, 74.4% anxiolytics, and other medications. Participants were randomized into either active anodal tDCS (*n* = 17) or sham stimulation (*n* = 22) targeting the left SI, with blinded stimulation codes. All were naive to the procedure and provided written informed consent. The study adhered to the Declaration of Helsinki (1991) and was approved by the Balearic Islands Ethics Committee (protocol IB3681/18PI).

### 2.2 Psychological assessment

A clinical psychologist obtained the participants´ clinical histories, medication use, sociodemographic data and psychological characteristics. The Spanish versions of the Beck’s Depression Inventory (BDI) (Beck et al., 1961) was used to quantify the severity of depressive symptoms. Current and habitual anxiety levels were quantified by the State–Trait Anxiety Inventory (STAI) (Spielberger et al., 1970). To evaluate alexithymia levels (i.e., the ability to identify, distinguish, and communicate their feelings) and the impact of the fibromyalgia symptoms in everyday life, the Toronto Alexithymia Scale (TAS-20) (Bagby et al., 1994) and the Fibromyalgia Impact Questionnaire (FIQ) (Burckhardt et al., 1991) were, respectively, used. The McGill Pain Inventory (Melzack, 1975) was administered to assess clinical pain. Finally, positive and negative affect was evaluated by the Manual for the Profile of Mood States (POMS) (McNair et al., 1971) before tDCS. The POMS measures six different dimensions of mood swings over a period of time, namely Tension or Anxiety, Anger or Hostility, Vigour or Activity, Fatigue or Inertia, Depression or Dejection, and Confusion or Bewilderment. In addition, participants reported their confidence and anxiety levels about the upcoming direct current stimulation using a visual-analogical scale (VAS-tDCS; from 0 to 10).

### 2.3 Transcranial electrical stimulation

The stimulation protocol followed a previously described double-blinded tDCS design (Montoro et al., 2021), using a NeuroConn constant-current stimulator (at 1.5 mA) and gel electrodes (20 mm diameter, 3 cm^2^ area, 1 mm thickness). The anode was placed at the left SI (CP3 location) and the cathode over the contralateral supraorbital ridge, following previous related papers (Rehmann et al., 2016; Friedrich and Beste, 2018). Active tDCS included a 20-min stimulation with 30-s ramp-up and ramp-down phases. The sham condition replicated active tDCS sensations with a brief current application (1.5 mA for 40 s) followed by no stimulation for the remainder of the session, maintaining electrode impedance below 5 kΩ. Participants sat in an armchair with eyes open during the session. Post-stimulation, they completed a questionnaire on transcranial electrical stimulation (TES)-related sensations (e.g., itching, pain, metallic taste) to assess bodily experiences, their duration, and effects on well-being. The protocol adhered to safety guidelines for tDCS implementation (Thair et al., 2017; Antal et al., 2017).

### 2.4 Non-painful paired-pulse stimulation task

As in prior studies of our research group (Montoya et al., 2006; Montoro et al., 2021; Terrasa et al., 2018), participants underwent tactile paired-pulse stimulation before and after electrical intervention to assess sensory gating. This involved two identical non-painful pneumatic stimuli (S1 and S2) lasting 100 ms each, with a randomized 550 ms (±50 ms) inter-stimulus interval. Each pair of stimuli was delivered with an inter-pair interval of 12 s. Stimulation was delivered via a pneumatic stimulator (2 bars pressure) through a 10-meter tube to a ductile membrane affixed to the index finger’s ventral first phalanx of the dominant (right) hand using a plastic clip and adhesive strip. Before starting, a tactile test ensured stimulus detection. Participants, instructed to keep their eyes open, received 40 trials administered with standard software (Presentation v18.3, Neurobehavioral Systems, Inc.) immediately before and after anodal tDCS or sham. Stimulus intensity was consistent (2 bars) across trials, and participants were asked to focus on the tactile sensations while seated comfortably with eyes open throughout the session.

### 2.5 EEG recording and data reduction

During the paired-pulse stimulation task, EEG recording was conducted using a commercial amplifier (QuickAmp, Brain Products GmbH, Munich, Germany) with 62 Ag/AgCl electrodes located according to the 10–10 placement system with common average reference, at a sampling rate of 1,000 Hz. The ground electrode was located at AFz. During EEG recording, eye blinks were recorded using an electrooculogram (EOG) placing one electrode above and another below the left eye. Electrode’s impedance was kept below 10 kOhm.

During offline data pre-processing, using Brain Vision Analyzer, version 1.05 software (Brain Products GmbH, Munich, Germany), EEG signals were segmented in epochs of 600 ms (−100 ms to 500 ms relative to the stimulus onset), filtered digitally (high-pass at 0.10 Hz, low-pass at 30 Hz), and baseline corrected (from −100 ms to 0 ms). Eye blink artifacts were corrected by using Gratton & Coles algorithm (Gratton et al., 1983). Artefact rejection was carried out using the following criteria: maximum voltage step/sampling point = 75 μV, minimum amplitude = −75 μV, maximum amplitude = 75 μV and a maximum absolute difference in the epoch = 75 μV. One participant from the active group did not meet the inclusion criteria for further analyses, which consisted of presenting at least 75% of the epochs free of artefacts for each stimulus. Therefore, 16 participants were included in the active group for EEG analyses. In addition, EEG epochs were separately averaged for S1 and S2.

The amplitudes of the following components of the SEPs were determined: P50, N100 and Late Positive Complex (LPC). The peak amplitude of P50 and N100 was calculated from the baseline for each individual channel within two-time windows after stimulus onset: 20–80 ms for P50, and 80–135 ms for N100. For LPC amplitudes, the area under the curve within the time period of 150–350 ms was calculated.

### 2.6 Procedure and experimental timeline

The study was conducted across two same-day sessions. In the first session, informed consent, sociodemographic, clinical data and pain ratings from a quantitative sensory testing (QST) were obtained. The QST consisted of three consecutive threshold measurements with a resting period between each measurement of 30 s. Afterwards, the TAS-20, STAI, BDI-II, FIQ, McGill Inventory, POMS, and VAS-tDCS were handed out to the participants. The second session (experimental task) was conducted after a brief 10-min break. During this session, participants were firstly accompanied to the EEG laboratory, where they sat comfortably in an armchair placed inside a Faraday chamber. Then, the experimenter mounted the electrodes for EEG recording and tDCS on the patients’ scalp. EEG recording started with the conduction of the non-painful Paired-Pulse Stimulation Task as described. After the completion of the task and a brief 2-min break, patients received 20 min of brain stimulation (anodal tDCS or sham), followed by another brief 2-min break. Finally, participants had to complete the TES and to perform the non-painful Paired-Pulse Stimulation Task.

### 2.7 Data analysis

All statistical analyses were carried out using IBM SPSS Statistics 29.0 (IBM Corp., Armonk, NY, USA). Group differences on sociodemographic data, medication use and self-reports were analyzed with Student t-tests. In order to analyze the SEPs response, EEG data from 30 electrodes (F1, F2, F3, F4, F5, F6, F7, F8, FC1, FC2, FC5, FC6, C1, C2, C3, C4, C5, C6, CP1, CP2, CP5, CP6, P1, P2, P3, P4, P5, P6, P7 and P8) were used for statistical analyses. A multivariate analysis of variance (MANOVA) with repeated measures was conducted, using group (tDCS vs. sham) as between-subject factor and time (pre vs. post), hemisphere (left vs. right) and electrode (30 electrodes) as within-subject factors on P50, N100, and LPC amplitude differences elicited by S1 minus S2 as sensory gating indicator. The level of significance was set at *p* ≤ 0.05 (2-tailed). Greenhouse–Geisser adjustments were applied and post-hoc Bonferroni corrected paired tests were used if necessary. Finally, although it was not one of the objectives of the present study, the effects of tDCS on S1 and S2 were analyzed independently. To achieve this, the same repeated measures MANOVA was conducted for S1 and S2 separately.

## 3 Results

### 3.1 Clinical and sociodemographic data

Table 1 shows clinical and sociodemographic data for both groups. No significant differences between groups were found in any of the variables analyzed (all *p* > 0.093).

**Table 1:** Clinical and sociodemographic data.

Measure category	Subcategory	tDCS (*n* = 17) Mean ± SD	sham (*n* = 22) Mean ± SD	*t*-value	*p*
Age (years)		55.12 ± 8.60	55.91 ± 7.42	−0.308	0.760
Medication use	Analgesics	17	18	1.893	0.066
	Anti-allergic	1	4	−1.129	0.266
	Antihistaminic	2	4	−0.539	0.593
	Antidepressants	15	19	0.169	0.867
	Sedatives	13	17	−0.057	0.955
	Anxiolytics	12	17	−0.463	0.646
	Contraceptives	3	2	0.778	0.441
BDI		26.06 ± 6.83	24.68 ± 12.01	0.451	0.655
STAI	Trait	27.29 ± 6.20	30.64 ± 6.18	−1.674	0.103
	State	22.88 ± 4.03	24.32 ± 8.24	−0.714	0.480
TA total		55.47 ± 8.36	55.23 ± 12.17	0.070	0.944
	DIF	20.53 ± 5.54	20.67 ± 7.22	−0.064	0.949
	DDF	13.24 ± 4.04	13.76 ± 4.03	−0.400	0.691
	EOT	21.71 ± 4.99	21.33 ± 4.27	0.248	0.805
Electrical stimulation	Confidence	8.50 ± 1.76	8.93 ± 1.50	−0.819	0.418
	Anxiety	2.44 ± 2.90	2.21 ± 3.33	0.225	0.823
FIQ		64.80 ± 17.60	69.00 ± 19.84	−0.688	0.496
McGill		78.70 ± 23.61	84.80 ± 16.92	−0.927	0.360
POMS total		146.24 ± 28.70	162.20 ± 50.60	−1.243	0.222
	Tension-anxiety	8.06 ± 5.90	10.70 ± 9.92	−1.028	0.311
	Depression-dejection	16.82 ± 10.70	23.60 ± 16.90	−1.517	0.138
	Anger-hostility	9.71 ± 6.80	12.27 ± 9.25	−0.961	0.343
	Vigour-activity	10.53 ± 7.20	9.50 ± 5.80	0.519	0.607
	Fatigue-inertia	12.70 ± 5.80	15.64 ± 7.30	−1.391	0.173
	Confusion-bewilderment	9.53 ± 4.20	13.00 ± 8.00	−1.732	0.093
TES	Itching	1.18 ± 1.01	1.05 ± 1.13	0.380	0.706
	Pain	0.18 ± 0.39	0.36 ± 0.79	−0.968	0.340
	Burning	0.41 ± 0.71	0.32 ± 0.78	0.390	0.699
	Warmth/Heat	0.47 ± 0.80	0.32 ± 0.57	0.666	0.511
	Metallic/Iron taste	0.18 ± 0.73	0.14 ± 0.47	0.198	0.845
	Fatigue/Decreased alertness	1.41 ± 1.12	1.36 ± 1.17	0.130	0.897

BDI = Beck’s Depression Inventory; STAI = State–Trait Anxiety Inventory; TA = Total Alexithymia (TAS-20); DIF = Difficulty Identifying Feelings (TAS-20); DDF = Difficulty Describing Feelings (TAS-20); EOT = External Oriented Thinking (TAS-20); FIQ = Fibromyalgia Impact Questionnaire; McGill = McGill Pain Questionnaire; POMS = Profile of Mood State Questionnaire; TES = Questionnaire of Sensations Related to Transcranial Electrical Stimulation.

### 3.2 Somatosensory ERP amplitudes

**Figure 1:**
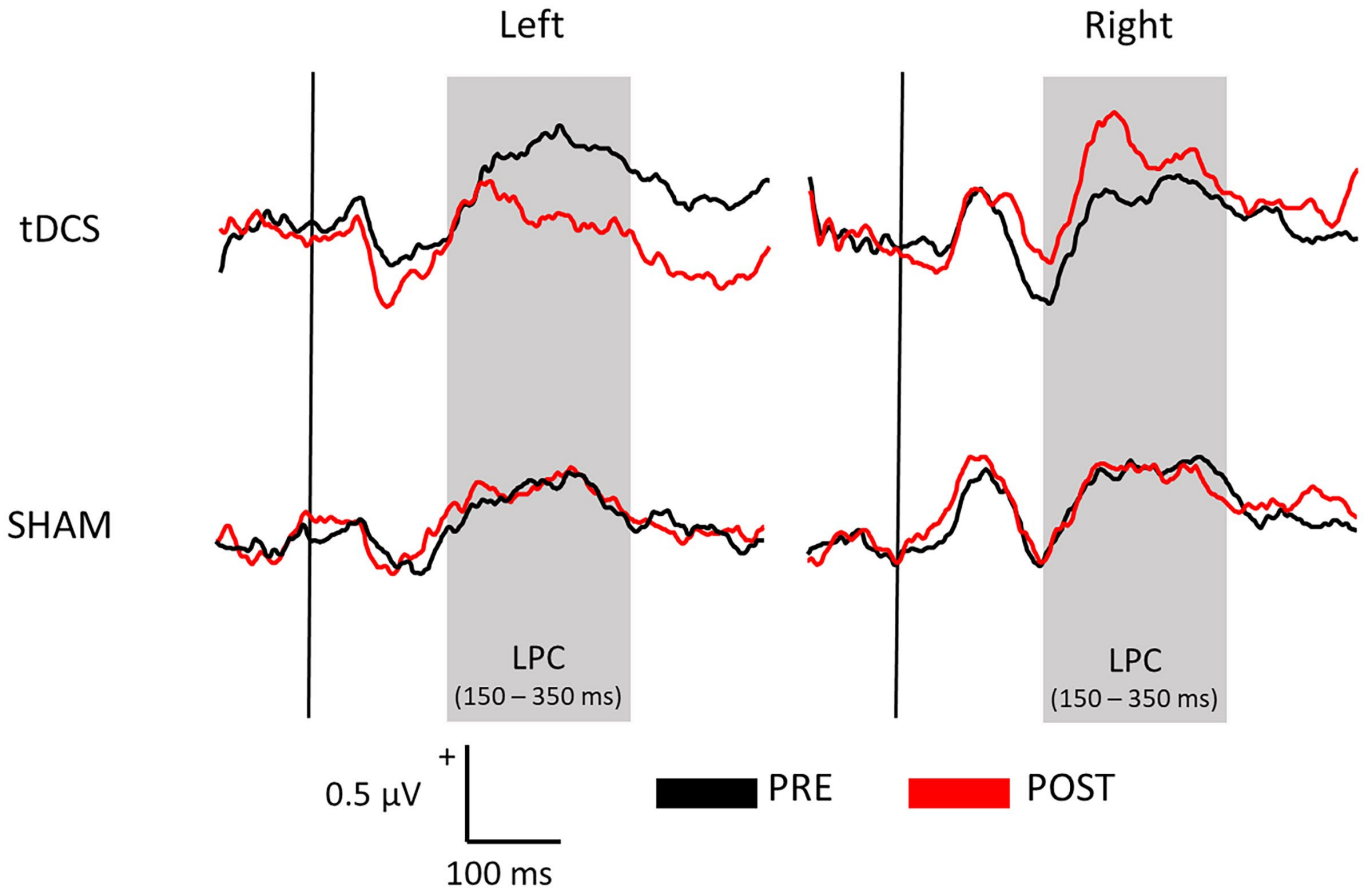
Average waveforms across all electrodes representing the sensory gating (S1 – S2) of both tDCS and SHAM groups before (PRE) and after (POST) the brain stimulation at each hemisphere.

**Figure 2:**
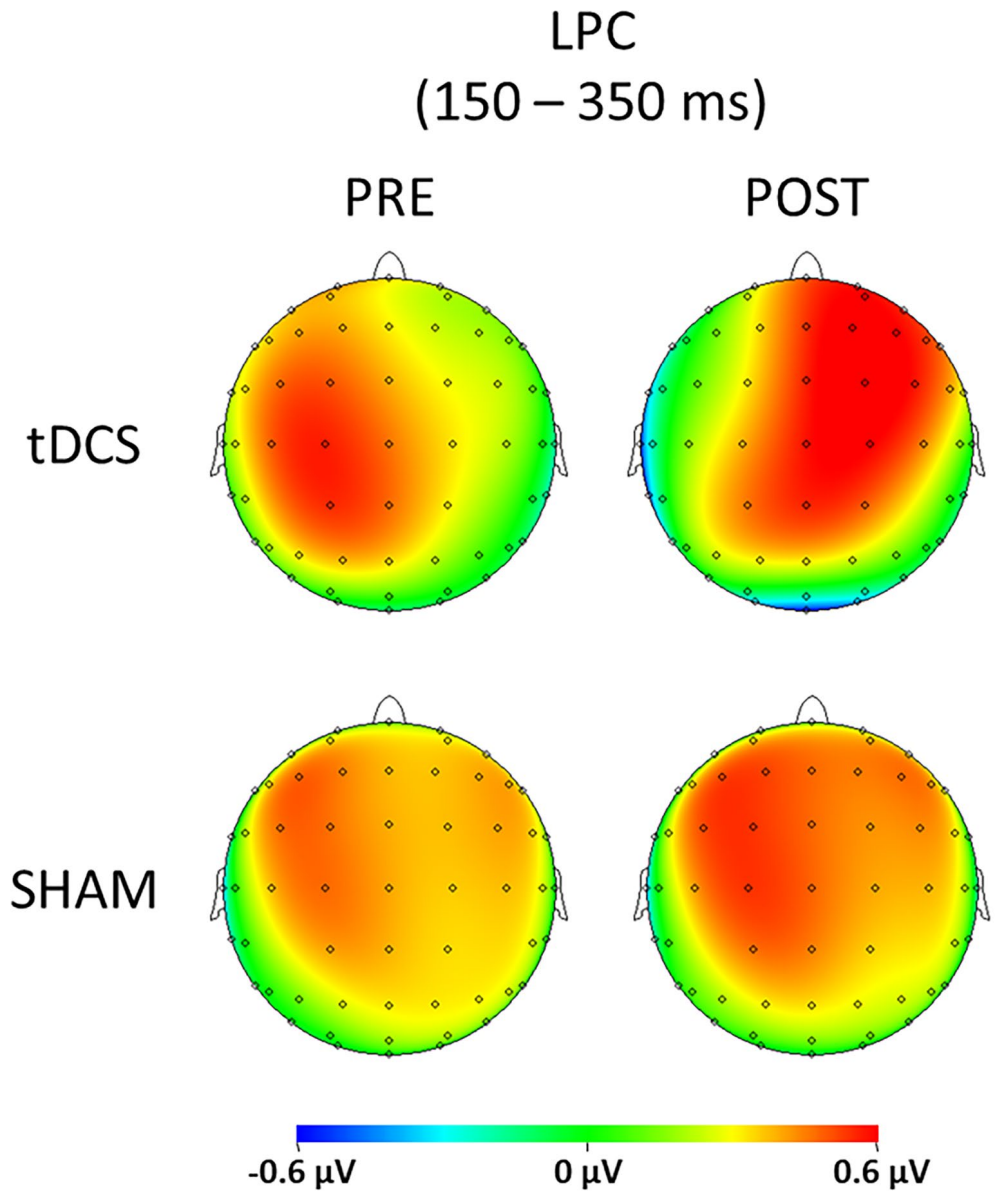
Topographic plots representing the sensory gating (S1 – S2) of both tDCS and SHAM groups before (PRE) and after (POST) the brain stimulation for the LPC component (150–350 ms).

Figure 1 displays the SEPs amplitude difference elicited by S1 minus S2 as sensory gating measure in each hemisphere. For LPC component, a significant interaction effect of group x time x hemisphere was found [*F*(1, 36) = 5.742, *p* = 0.022, ŋp^2^ = 0.138], showing that sensory gating was significantly enhanced after stimulation (88.49 ± 19.90 μV*ms) compared with before (39.03 ± 17.75 μV*ms) at right hemisphere electrodes (*p* = 0.043) in the experimental group (tDCS group) (see Figure 2 for topographical plots). Surprisingly, an inverse significant effect was found at left hemisphere electrodes (*p* = 0.009), as sensory gating was reduced after stimulation (15.80 ± 26.93 μV*ms) compared with before (77.12 ± 19.97 μV*ms) (see Table 2). No significant effects were obtained in the sham group at any hemisphere (all *p* > 0.637). The remaining main effects and interactions can be observed in Table 3.

**Table 2:** Mean (±SE) of the sensory gating effect (S1 minus S2) in the tDCS and sham groups, before (PRE) and after (POST) stimulation at each hemisphere.

Group	SEP component	Hemisphere	PRE	POST	*p*
tDCS (*n* = 16)	P50 (μV)	Left	0.21 ± 0.07	−0.05 ± 0.11	0.534
		Right	0.27 ± 0.12	0.28 ± 0.15	0.942
	N100 (μV)	Left	−0.16 ± 0.09	−0.29 ± 0.16	0.407
		Right	−0.37 ± 0.11	−0.21 ± 0.10	0.080
	LPC (μV*ms)	Left	77.12 ± 19.97	15.80 ± 26.93	**0.009**
		Right	39.03 ± 17.74	88.49 ± 19.90	**0.043**
Sham (*n* = 22)	P50 (μV)	Left	0.15 ± 0.06	0.12 ± 0.09	0.771
		Right	0.34 ± 0.10	0.48 ± 0.13	0.186
	N100 (μV)	Left	−0.16 ± 0.08	0.07 ± 0.14	0.105
		Right	−0.11 ± 0.09	−0.07 ± 0.08	0.611
	LPC (μV*ms)	Left	45.11 ± 17.03	54.12 ± 22.97	0.637
		Right	61.96 ± 15.13	63.93 ± 16.97	0.922

Bold values indicate significance at *p* < 0.05 and *p* < 0.01.

**Table 3:** Results of the multivariate analysis of variance (MANOVA) with repeated measures for LPC component, indicating degrees of freedom (DF), test statistic value (*F*), significance level (*p*), and effect size (*ŋp^2^*).

Interactions	DF	*F*	*p*	*ŋp^2^*
time	1, 36	0.001	0.980	0.000
time * GROUP	1, 36	0.431	0.516	0.012
hemisphere	1, 36	0.701	0.408	0.019
hemisphere * GROUP	1, 36	0.012	0.914	0.000
electrode	14, 504	4.453	**0.007**	0.110
electrode * GROUP	14, 504	0.619	0.592	0.017
time * hemisphere	1, 36	4.453	**0.042**	0.110
time * hemisphere * GROUP	1, 36	5.742	**0.022**	0.138
time * electrode	14, 504	0.528	0.736	0.014
time * electrode * GROUP	14, 504	1.188	0.318	0.032
hemisphere * electrode	14, 504	5.803	**0.001**	0.139
hemisphere * electrode * GROUP	14, 504	0.806	0.501	0.022
time * hemisphere * electrode	14, 504	3.337	**0.011**	0.085
time * hemisphere * electrode * GROUP	14, 504	1.412	0.231	0.038

*denotes interaction terms between factors in the repeated measures MANOVA. Bold values indicate significance at *p* < 0.05 and *p* < 0.01.

However, no significant effects or interactions were obtained for P50 and N100 components. Finally, the repeated measures MANOVA was also conducted for S1 and S2 independently. Results are depicted in the Supplementary material.

## 4 Discussion

The present study analyzed for the first time the after-effects of anodal tDCS on brain correlates of somatosensory inhibitory mechanisms in FMS patients. Considering that FMS is characterized by a lack of inhibitory control to repetitive non-painful somatosensory information, the possible modulatory effects of somatosensory gating caused by anodal tDCS was explored by using somatosensory event-related potentials elicited by paired tactile stimuli. Previous studies have shown that brain responses to somatosensory gating were significantly altered in FMS (Montoya et al., 2006). In the same way, previous work has demonstrated that non-invasive brain stimulation techniques such as anodal tDCS can induce significant improvements in the symptomatology of chronic pain patients such as FMS (Pacheco-Barrios et al., 2020; Pinto et al., 2018). The present study demonstrates that anodal tDCS induces significant modulation of brain processing. Specifically, our results revealed that tDCS altered late somatosensory responses during the gating paradigm. Notably, anodal tDCS enhanced the S1-S2 difference—the parameter used to index sensory gating—over the right hemisphere (contralateral to the stimulation site), while, unexpectedly, it reduced this difference over the left hemisphere (ipsilateral to the stimulation site). As expected, no changes in the difference S1-S2-and therefore gating-were observed in the sham group.

Our findings were partially in agreement with previous results in healthy controls indicating that anodal tDCS was associated with a significant amplitude enhancement of early SEP components (<100 ms) elicited by electrical stimulation of the body (Matsunaga, 2004). Previous studies have also reported a beneficial effect of tDCS on auditory hallucinations and negative symptoms, as well as an improvement of sensory gating in the early stages of auditory information processing (P50, N100) in patients with schizophrenia (Kim et al., 2018; Mondino et al., 2016). In line with these findings, our results revealed that anodal tDCS in FMS patients also produced a significant modulatory effect on somatosensory gating, as indicated by changes in the S1–S2 difference of the LPC component. After the stimulation, a widespread enhanced S1-S2 difference—suggestive of a sensory gating effect—was observed in the right hemisphere (contralateral to the place where tDCS was applied). LPC is a positive ongoing response consisting of different independent components, with a peak around 300 ms after stimulus-onset (Rushby et al., 2005). Mainly, LPC amplitudes of event-related potentials are positively correlated to the increase in cognitive demand and the situation’s personal significance, whereas LPC latency seems to be independent of stimulus classification and evaluation time (Rushby et al., 2005; De Pascalis, 2004). Considering the latter, the present results suggested that tDCS may optimally modulate late and cognitively more demanding stages of somatosensory processing in FMS patients, thus improving the altered ability to inhibit repetitive stimuli in FMS (in particular, over contralateral regions to the stimulated brain area). This finding is also in agreement with a previous study from our lab in healthy volunteers (Montoro et al., 2021), showing that anodal tDCS led to an enhancement of inhibitory mechanisms in response to repetitive somatosensory stimulation during late stages of information processing.

However, we observed that brain stimulation impaired the S1-S2 difference—reflecting a disruption of the somatosensory gating effect—of LPC over the left hemisphere (ipsilateral to anode stimulation). This inverse pattern of tDCS modulation (as compared to the right hemisphere) is difficult to explain, and it could be related not only with the tDCS neural target (left primary somatosensory cortex), but also with the tactile stimulation site itself (right hand forefinger). To this regard, most of the studies with FMS patients have stimulated the left primary motor cortex (M1) or the left dorsolateral prefrontal cortex (DLPFC). M1 has been proposed to be the most effective in modulating cortex excitability and pain sensory processing in FMS patients while DLPFC would have greater effects on emotional symptomatology and affective-cognitive pain processing (Cummiford et al., 2016; Lorenz et al., 2003). Furthermore, although the observed hemispheric asymmetry may be partly attributed to the site of stimulation and the tactile input, lateralized attentional mechanisms might also have contributed (Shulman et al., 2010; Wang et al., 2024). However, spatial attention was not explicitly manipulated in our paradigm, as all stimuli were delivered to the same body location while participants maintained a fixed gaze and posture. Since the modulation emerged only in the experimental group and was confined to the LPC component, with no changes observed in early components (P50, N100), the effect is more likely to reflect higher-order evaluative or integrative processing rather than early attentional orienting. Given the present results, similar comparative studies-and in general, studies delving into the present findings-are required for a better comprehension of the effects of non-invasive brain stimulation on somatosensory information processing in FMS. A bilateral anodal stimulation in both SI cortices could match the pattern toward a generalized improvement of somatosensory gating. Cathodal stimulation (inhibition) rather than anodal stimulation (activation) in patients with FMS, could also stand for the reversion of the observed gating deterioration on the ipsilateral stimulated hemisphere (Vaseghi et al., 2015).

In contrast with previous studies in healthy participants (Beck et al., 1961), the lack of modulatory effects on the difference S1-S2, and therefore, somatosensory gating of early stages of information processing in our FMS participants remains unclear. One possible explanation is that early stages of information processing (such as those mirrored by P50 and N100 amplitudes) of non-painful stimulation are more difficult to modulate using one session of non-invasive electrical brain stimulation in FMS. In this sense, P50 has been associated with the early coding and processing of non-painful stimuli (Desmedt and Tomberg, 1989; Staines et al., 2014). The impaired P50 sensory gating observed in FMS may reflect a less effective filtering of sensory information, indicating an alteration in early-stage cognitive processing mechanisms (Kofler and Halder, 2014). Indeed, former research suggested the impairment in early attenuation of repetitive sensory input in chronic pain patients as a marker of a generalized deficit on multisensory inhibition (Montoya et al., 2006; Ambrosini et al., 2001; Carrillo-De-La-Peña et al., 2015; Siniatchkin et al., 2003). Regarding the N100 component, it has been suggested to be involved in the triggering of attention and early perceptual processing (Shen et al., 2020). It has also been linked to central integrative processes and is widely considered a marker of attentional modulation (Giard et al., 1988; Näätänen and Picton, 1987). N100 abnormalities in FMS patients are assumed to reflect deficient sensory encoding and/or registration (Choi et al., 2016). Thus, it seems that a single tDCS session might not be sufficient to modify the deep alterations in early stages of sensory gating that characterize a chronic pain condition, such as FMS. Congruently, a meta-analysis demonstrated larger tDCS effects in FMS patients using protocols that lasted four weeks or more (Teixeira-Santos et al., 2022). Alternatively, it could be that auditory and electrical stimulation, as those used in previous studies, could be more powerful sensory modalities for eliciting changes in gating mechanisms after non-invasive brain stimulation (Matsunaga, 2004; Kim et al., 2018). Finally, it is important to reiterate that in our previous study with healthy participants (Montoro et al., 2021), we also did not observe any modulation of sensory gating in early components. Therefore, it is possible that the combination of anodal stimulation over the SI and tactile stimulation to generate gating, may be responsible for the reported results.

Several limitations and future directions in forthcoming studies should not be overlooked. First, it is known that certain parameters substantially influence tDCS outcomes, e.g., electrode montage, stimulation intensity and duration, and stimulation protocol (Jürgens et al., 2012; Kunzelmann et al., 2018). Therefore, the present results must be considered with caution before drawing any firm conclusion about the efficacy of anodal tDCS on sensory gating in FMS patients; as differences between our study results and previous research could be attributable to distinct stimulation protocols and study design. Second, the findings of this study might be biased by the small sample size. Third, the inclusion of 30 electrodes in the statistical analyses increases the risk of type I error and may reduce spatial specificity, which should be addressed in future studies through more targeted region-of-interest or data-driven clustering approaches. Fourth, all our participants were taking pharmacological medication. Although there were no significant differences in medication use between groups, the role of medication in the observed brain activity cannot be completely ruled out. Many participants were under medications such as analgesics or antidepressants, which can alter cortical excitability and affect electrophysiological responses (Minzenberg and Leuchter, 2019). These medications might have modulated baseline neural activity or interacted with tDCS-induced changes, potentially confounding the interpretation of sensory gating effects. Due to the sample size and study design, controlling for medication effects statistically was not feasible. Future studies should aim to include medication-free patients or control for medication variables more rigorously to clarify their impact on neurophysiological outcomes in FMS research. Fifth, it is important to highlight that although the understanding of the electrophysiological effects of tDCS has progressed over the last years, its precise mechanisms of action still have to be unveiled (Chase et al., 2020; Yamada and Sumiyoshi, 2021). TDCS has not only been demonstrated to cause diffuse and widespread effects across cortical functional networks (Bestmann et al., 2015) but also these effects have been shown to depend on the resting-state of the brain at the moment of stimulation (Woods et al., 2016). Furthermore, even though it was ensured that all participants were given the same instructions to rest and relax at the initial adaptation phase, it was not possible to control for the individuals´ expectations, beliefs or thoughts; factors that are also known to influence tDCS outcomes (Gill et al., 2015; Segrave et al., 2014). The absence of a healthy control group also limits the generalization of findings exclusively to the FMS population. To address these limitations, a within-subject crossover design including both active anodal tDCS and sham stimulation, as well as healthy controls, is strongly recommended for future studies. This approach would better control for individual differences, placebo effects, and disease-specific responses, thereby enhancing the reliability and validity of conclusions about tDCS effects on somatosensory processing in FMS patients.

Finally, it is important to acknowledge that only a single tDCS session was applied. While this design was appropriate for exploring fundamental neurophysiological mechanisms, clinical effects of tDCS typically emerge after multiple sessions. Moreover, as we did not examine correlations between LPC sensory gating changes and clinical characteristics such as symptom severity, our conclusions regarding long-term or therapeutic efficacy are limited. The observed changes in LPC amplitudes reflect modulation of late-stage somatosensory processing but represent a neurophysiological effect whose clinical significance remains uncertain. Since LPC is not a direct marker of symptom improvement and its translational relevance to therapeutic outcomes in FMS remains to be established, these findings should be interpreted as evidence of cortical modulation rather than clinical benefit. Future studies integrating both neurophysiological and clinical measures will be crucial to determine the potential therapeutic value of tDCS-induced sensory gating modulation.

## 5 Conclusion

A single and short (20 min.) session of anodal tDCS (1.5 mA) elicited a significant modulation of the S1-S2 difference, employed as a functional marker of the somatosensory gating process, in FMS. This effect was mirrored by an enhancement of S1-S2 difference—indicating increased inhibition to repetitive somatosensory stimulation—over the right hemisphere (contralateral to the stimulation), and by a reduction of this difference—indicating diminished inhibition—over the left hemisphere. Moreover, we observed that these effects appeared in the later stages of the somatosensory brain response, such as LPC amplitudes, but not in the early brain responses, such as P50 and N100 amplitudes. Thus, it seemed that the effects of anodal tDCS in FMS was mainly restricted to cognitive evaluation, and not to the coding and perceptual processing of bodily information. Importantly, these effects reflect a modulation of cortical processing, but their direct relevance to clinical improvement remains to be determined. Furthermore, given that clinical effects of tDCS seem to appear after several sessions, our findings suggest the need to explore the modulatory effects of tDCS (and other non-invasive brain stimulation techniques) in somatosensory processing after multiple sessions and follow-up sessions. Such research should incorporate both neurophysiological and clinical endpoints to determine whether repeated stimulation can contribute to reversing the maladaptive plasticity often associated with chronic pain syndromes. Finally, future studies should be conducted to explore the effects of both anodal and cathodal brain stimulation aimed at reversing the alterations in brain activity commonly observed in FMS patients.

## Data Availability

The raw data supporting the conclusions of this article are held by the first author and will be made available upon request, without undue reservation.
