# Correction: Modulatory effects of transcranial direct current stimulation on sensory gating in Fibromyalgia Syndrome

**DOI:** 10.3389/fpsyg.2026.1832608

**Published:** 2026-04-29

**Authors:** Juan L. Terrasa, Christine Winterholler, Pedro Montoya, Antonio Juan, Casandra I. Montoro

**Affiliations:** 1 Cognitive and Affective Neuroscience and Clinical Psychology, Research Institute of Health Sciences (IUNICS) and Balearic Islands Health Research Institute (IdISBa), University of the Balearic Islands (UIB), Palma, Spain; 2 Rheumatology, Hospital Universitari Son Llàtzer, Palma de Mallorca, Spain; 3 Department of Psychology, University of Jaén, Jaén, Spain

**Keywords:** transcranial direct current stimulation, somatosensory cortex, sensory gating, late positive complex, Fibromyalgia Syndrome

An incorrect **Funding** statement was provided in the published article. The correct **Funding** statement reads:

The author(s) declare that financial support was received for the research and/or publication of this article. This work was supported by grants (#PSI2017-88388-C4-1-R, #PID2022-140561NB-I00) from the Spanish State Research Agency (MICIU/AEI/10.13039/501100011033) and the European Regional Development Funds (ERDF), a competitive contract (Juan de la Cierva-Formación, FJCI-2016-29088) funded by the Ministry of Science, Innovation and Universities, the State Research Agency and the University of the Balearic Islands (author CM), and a competitive postdoctoral contract (PostDoc: Margalida Comas, PD/018/2021) funded by Conselleria de Fons Europeus, Universitat i Cultura of the Balearic Islands Government and the University of the Balearic Islands (author JT).

The original version of this article has been updated.

