# Supplementary material for: Modulatory effects of transcranial direct current stimulation on sensory gating in Fibromyalgia Syndrome
Source: Front Psychol. 2025 Aug 19;16:1607317. doi: 10.3389/fpsyg.2025.1607317 (PMC12402938; doi:10.3389/fpsyg.2025.1607317)

***Supplementary Material***

# Supplementary Data

**Repeated measures MANOVA of S1 and S2 independently, for each component.**

First stimuli (**S1**): for **P50**, a main effect of *hemisphere* [*F*(1,36) = 15.824, *p* = 0.000, *ŋp²* = 0.300] was found. Hence, no pre-post differences in S1 were identified in the experimental group. For **N100**, significant interaction effects of *hemisphere x electrode* [*F*(14,504) = 4.242, *p* = 0.004, *ŋp²* = 0.103] and *group x time x hemisphere* [*F*(1,36) = 5.929, *p* = 0.020, *ŋp²* = 0.138] were found. However, no pre-post differences in S1 were found in the experimental group. For **LPC**, a main effect of *electrode* was found [*F*(14,504) = 3.159, *p* = 0.029, *ŋp²* = 0.079] and significant interaction effects of *hemisphere x electrode* [*F*(14,504) = 8.194, *p* = 0.000, *ŋp²* = 0.181], *time x hemisphere x electrode* [*F*(14,504) = 2.703, *p* = 0.013, *ŋp²* = 0.068], *group x time x hemisphere* [*F*(1,36) = 4.458, *p* = 0.042, *ŋp²* = 0.108] and *group x time x hemisphere x electrode* [*F*(14,504) = 3.385, *p* = 0.003, *ŋp²* = 0.084] were found. However, no pre-post differences in S1 were found in the experimental group for LPC.

Second stimuli (**S2**): for **P50**, a main effect of *hemisphere* [*F*(1,36) = 13.031, *p* = 0.001, *ŋp²* = 0.260] and *electrode* [*F*(14,504) = 6.147, *p* = 0.004, *ŋp²* = 0.142] were found. For **N100**, a main effect of *time* [*F*(1,36) = 4.534, *p* = 0.040, *ŋp²* = 0.106] and *hemisphere* [*F*(1,36) = 4.879, *p* = 0.033, *ŋp²* = 0.117], and significant interaction effect of *hemisphere x electrode* [*F*(14,504) = 4.196, *p* = 0.002, *ŋp²* = 0.102] were found. For **LPC**, significant interaction effects of *time x hemisphere* [*F*(1,36) = 5.816, *p* = 0.021, *ŋp²* = 0.136] and *hemisphere x electrode* [*F*(14,504) = 2.619, *p* = 0.021, *ŋp²* = 0.066] were found. Hence, no pre-post differences in S2 were identified in the experimental group for P50, N100 or LPC.

# Supplementary Figures

**Supplementary Figure 1**. Average waveforms across all electrodes representing the first somatosensory stimuli (S1) of both tDCS and SHAM groups before (PRE) and after (POST) the brain stimulation at each hemisphere.


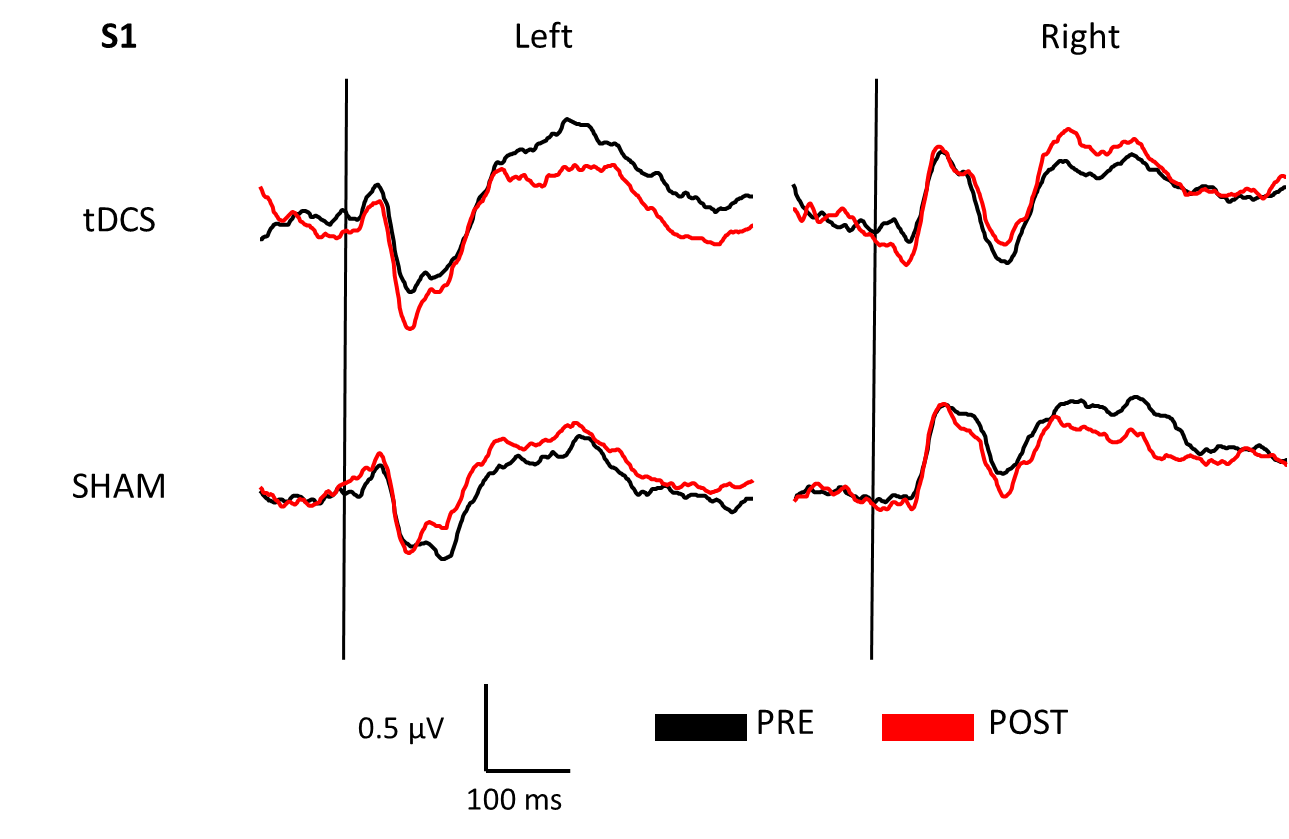


**Supplementary Figure 2.** Average waveforms across all electrodes representing the second somatosensory stimuli (S2) of both tDCS and SHAM groups before (PRE) and after (POST) the brain stimulation at each hemisphere.


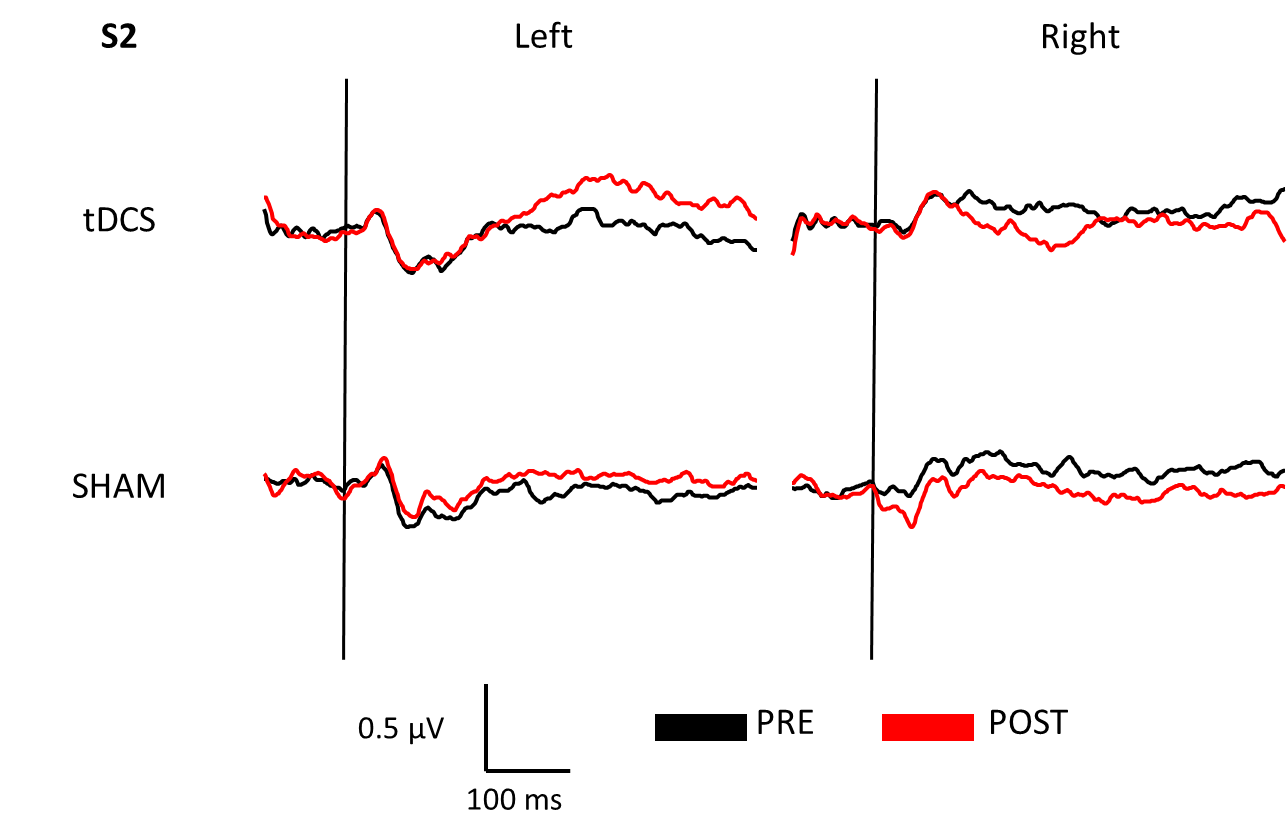

Supplement: Supplementary file 1 [file Supplementary_file_1.docx]
